# Supplementary material for: Albumin-corrected anion gap as a predictive marker for mortality in critically Ill cirrhosis patients: an analysis based on the MIMIC-IV database
Source: PLoS One. 2025 Sep 12;20(9):e0332490. doi: 10.1371/journal.pone.0332490 (PMC12431220; doi:10.1371/journal.pone.0332490)
Supplement: S1 Table — Results of the sensitivity analysis. (DOCX) [file pone.0332490.s001.docx]

**Supplementary Table1**

Association between ACAG and mortality in patients with cirrhosis

| **Outcome** | Unadjusted HR (95%CI) | Model1 HR (95%CI) | Model2 HR (95%CI) |
| --- | --- | --- | --- |
| **30-d** | | | |
| ACAG≤18.375 | 1 | 1 | 1 |
| ACAG＞18.375 | 2.39(2.08, 2.75) | 2.40(2.09, 2.76) | 1.64(1.41, 1.91) |
| P | <0.001 | <0.001 | <0.001 |
| **90-d** | | | |
| ACAG≤18.375 | 1 | 1 | 1 |
| ACAG＞18.375 | 2.26(1.99, 2.57) | 2.27(1.99, 2.58) | 1.51(1.31, 1.75) |
| P | <0.001 | <0.001 | <0.001 |
| **180-d** | | | |
| ACAG≤18.375 | 1 | 1 | 1 |
| ACAG＞18.375 | 2.27(1.99, 2.58) | 2.27(2.00, 2.59) | 1.52(1.32, 1.75) |
| P | <0.001 | <0.001 | <0.001 |
| **365-d** | | | |
| ACAG≤18.375 | 1 | 1 | 1 |
| ACAG＞18.375 | 2.27(2.00, 2.58) | 2.28(2.00, 2.59) | 1.53(1.32, 1.76) |
| P | <0.001 | <0.001 | <0.001 |

Model 1: Adjusted age and Gender.

Model 2: Model 1+ Temperature, Platelet, WBC, Sodium, INR, BUN, Sepsis, Variceal bleeding, SBP, Ascite, HRS, HE, AKI, and CRRT.
