# Supplementary figures and images for: Albumin-corrected anion gap as a predictive marker for mortality in critically Ill cirrhosis patients: an analysis based on the MIMIC-IV database
Source: PLoS One. 2025 Sep 12;20(9):e0332490. doi: 10.1371/journal.pone.0332490 (PMC12431220; doi:10.1371/journal.pone.0332490)

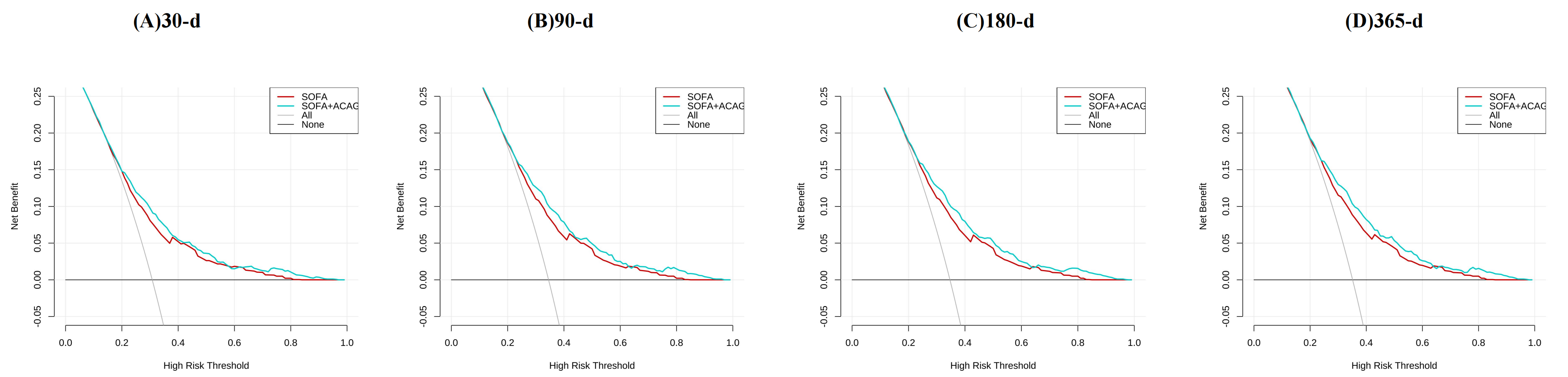

Supplement: S1 Fig — DCA curves for SOFA versus SOFA+ACAG in patients with cirrhosis at 30-d (A), 90-d (B), 180-d (C), and 365-d (D) of hospital admission. (TIF) [file pone.0332490.s004.tif]

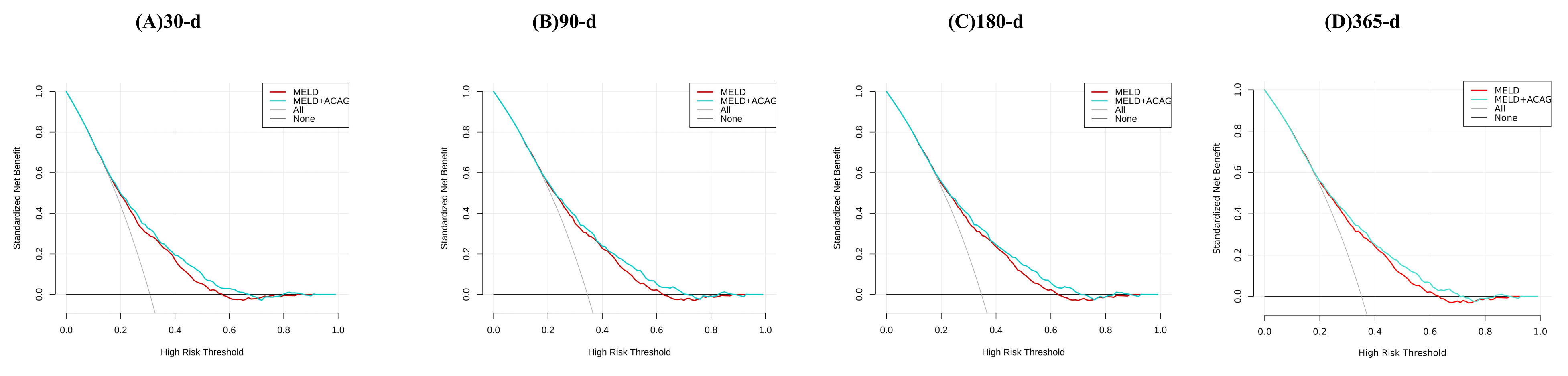

Supplement: S2 Fig — (TIF) [file pone.0332490.s005.tif]
