# Supplementary material for: Albumin-corrected anion gap as a predictive marker for mortality in critically Ill cirrhosis patients: an analysis based on the MIMIC-IV database
Source: PLoS One. 2025 Sep 12;20(9):e0332490. doi: 10.1371/journal.pone.0332490 (PMC12431220; doi:10.1371/journal.pone.0332490)
Supplement: Attachment — Submitted filename: Response to Reviewers.docx [file pone.0332490.s007.docx]

Dear Editor and Reviewers,

We sincerely thank you for giving us the opportunity to revise our manuscript titled **“Albumin-Corrected Anion Gap as a Predictive Marker for Mortality in Critically Ill Cirrhosis Patients: An Analysis Based on the MIMIC-IV Database**”. We greatly appreciate the time and effort both the editor and reviewers have dedicated to reviewing and suggesting improvements to our work. Truly, these invaluable comments and recommendations have assisted us in enhancing the quality of our manuscript. In response, we have made the necessary revisions. We have uploaded two versions of the manuscript: a "Revised Manuscript with Track Changes" and a "Cleaned Revised Manuscript". In the "Revised Manuscript with Track Changes" version, we have highlighted the changes in red for easy identification. Enclosed within this submission, we've provided a point-by-point response to each of the comments raised. The comments from the editor or reviewers are presented in blue italics. We genuinely hope that with these modifications, our manuscript now aligns with the standards of your esteemed journal.

### Required Revisions for Acceptance:

**Reviewer**

*1.Clarification of Cirrhosis Diagnosis (ICD Codes)*

*-Please explicitly list the ICD-9/10 codes used to define cirrhosis in the Methods section (currently not specified).*

**The author's response:**

We sincerely appreciate your valuable feedback. We acknowledge this oversight and have now included the relevant ICD code in the Methodology section ICD-9codes：571.2,571.5,571.6. ICD-10codes：K70.30, K70.31, K74.3, K74.60, K74.69.

***Cleaned*** ***Revised Manuscript, Page 5, lines 112-114:***

The diagnosis of cirrhosis was established based on the International Classification of Diseases (ICD) codes (ICD-9: 571.2, 571.5, 571.6; ICD-10: K70.30, K70.31, K74.3, K74.60, K74.69). Our study included 6597 patients with cirrhosis requiring ICU admission. We excluded patients with: (1) non-first ICU admissions; (2) ICU stays shorter than 24 hours; or (3) unavailable AG or albumin (ALB) data within 24 hours of admission. The final analysis included 2826 patients. **Fig 1** illustrates the study’s inclusion and exclusion process.

*2.Handling of Missing Data*

*-Expand on your current explanation of missing data. You mention Random Forest imputation for≤20% missingness—please justify this choice and specify which variables were imputed.*

**The author's response:**

Thank you for your insightful comments. Regarding your query on the imputation method, we selected the random forest imputation method (MissForest) for variables with a missing rate of ≤20%. We made this choice because MissForest has significant advantages over traditional methods, such as mean/median imputation or multiple imputation by chained equations (MICE). Traditional methods have limitations when dealing with complex data structures. For instance, mean/median imputation assumes that data are missing completely at random and cannot capture nonlinear relationships between variables. Although MICE improves on these limitations to some extent, it can still face challenges when handling high - dimensional data or complex correlations. Specifically: (1) The non - parametric adaptability of random forest imputation enables it to model complex nonlinear relationships effectively without requiring normality assumptions [1]. (2) Unlike regression - based imputation methods, random forest imputation is robust to outliers[2], allowing for a more accurate estimation of missing values.

[1]	Tang F, Ishwaran H. Random Forest Missing Data Algorithms. Stat Anal Data Min. 2017. 10(6): 363-377.

[2]	Stekhoven DJ, Bühlmann P. MissForest--non-parametric missing value imputation for mixed-type data. Bioinformatics. 2012. 28(1): 112-8.

***Cleaned*** ***Revised Manuscript, Page 7-8, lines 151-155:***

Data with >20% missing values were excluded; those with ≤20% underwent random forest imputation. Random forest imputation is a powerful tool for data completion. Its non-parametric nature enables it to model complex nonlinear relationships in data without strict normality assumptions[19]. Also, compared to regression-based methods, it shows strong robustness to outliers, improving the accuracy of missing value estimation[20].

*-please justify this choice and specify which variables were imputed.*

2. Variables Imputed and Missingness Patterns Imputation was applied to the following variables (missingness rates in parentheses):

Missing data distribution

| Heart rate | 0.2% |
| --- | --- |
| systolic blood pressure | 11.6% |
| diastolic blood pressure | 11.6% |
| MBP | 11.7% |
| Respiratory rate | 0.15% |
| Temperature | 3.7% |
| Hemoglobin | 0.2% |
| Platelet | 0.2% |
| RDW | 0.4% |
| RBC | 0.1% |
| WBC | 0.1% |
| TCa | 0.3% |
| Glucose | 0.1% |
| INR | 2.1% |
| ALT | 1.8% |
| AST | 1.4% |
| Total bilirubin | 1.4% |
| MELD | 3.4% |

*3.Normality and Data Presentation*

*-Clarify whether tests for normality (e.g., Shapiro-Wilk) were performed. If data were not normally distributed, justify the use of mean ± SD over median (IQR) for continuous variables.*

**The author's response:**

We sincerely appreciate your insightful comments regarding the statistical methodology. As suggested, we have carefully re-examined the statistical descriptions and made the following corrections: The normality of continuous variables was evaluated using Shapiro-Wilk tests. Normally distributed data were presented as mean ± standard deviation (SD) and analyzed with independent samples t-tests, while non-normally distributed variables were expressed as median (interquartile range, IQR) and analyzed using Mann-Whitney U tests.

***Cleaned*** ***Revised Manuscript, Page 7, lines 146-150:***

The normality of continuous variables was evaluated using Shapiro-Wilk tests. Normally distributed data were presented as mean ± standard deviation (SD) and analyzed with independent samples t-tests, while non-normally distributed variables were expressed as median (interquartile range, IQR) and analyzed using Mann-Whitney U tests. Data with >20% missing values were excluded; those with ≤20% underwent random forest imputation.

*4.Multivariate Model Construction*

*-Clearly describe the variable selection process for Model 2. State whether stepwise selection, expert judgment, or univariate p-values were used. Also confirm whether multicollinearity (e.g., using VIF) was assessed.*

**The author's response:**

We sincerely appreciate your valuable feedback.

1. Initial variable selection was based on statistically significant associations (P<0.05) in baseline characteristics analysis, including laboratory parameters (temperature, platelet count, WBC, sodium, INR, BUN), clinical complications (sepsis, spontaneous bacterial peritonitis, ascites, hepatorenal syndrome, hepatic encephalopathy, acute kidney injury), and therapeutic interventions (CRRT). Given the unique prognostic considerations in cirrhosis, clinically critical variables (e.g., variceal bleeding, P>0.05) that did not reach statistical significance in baseline analyses were retained based on their established clinical relevance. All selected variables underwent dual validation through (1) pathophysiological justification (e.g., INR reflecting coagulation status, BUN indicating renal function) and (2) support from clinical practice guidelines and expert consensus. The final predictive model incorporated three categories of variables: validated prognostic markers (assessing infection, liver function and disease severity), key complications, and therapeutic interventions (including CRRT).

2. All covariates were assessed for multicollinearity, and the diagnostic plot is presented below.

T-: temperature; V.B: variceal bleeding.

*5.Code and Dataset Availability*

*-Upload all relevant R code(particularly for ROC analysis, multivariate models, RCS plots, and subgroup analysis) and a de-identified dataset (in CSV format) as supplementary material. These are needed for transparency and reproducibility*.

**The author's response:**

Thank you for your valuable suggestion. As requested, I have included the relevant R scripts in the Supplementary Materials: 'ROC_analysis.R' (for ROC curve analysis), 'multivariate_models.R' (for multivariate modeling), 'subgroup_analysis.R' (for subgroup analyses), and 'RCS.R' (for restricted cubic spline modeling). The dataset has also been converted to CSV format and uploaded as supplementary material. I fully agree that research transparency and reproducibility are paramount, and I appreciate your insightful recommendation.

***Cleaned*** ***Revised Manuscript, Page 5, lines 109-110:***

The author accessed the database after completing the CITI Program (certificate #67058598). De-identified data can be found in the supplementary material “database”.

***Cleaned*** ***Revised Manuscript, Page 8, lines 174-175:***

All statistical analyses were performed using R software (version 4.2.2, R Foundation for Statistical Computing, Vienna, Austria) and MSTATA software ([https://www.mstata.com/](https://www.mstata.com/)). The code used is available in the supplementary material “R.zip”.

*6.Subgroup Analysis: Interaction Testing*

*-Clarify whether formal tests for interaction were conducted (i.e., inclusion of interaction terms in Cox models). If not, please either perform and report p-values for interaction or state explicitly why these were not included.*

**The author's response:**

Thank you for your valuable comment regarding our subgroup analysis. In response to your question, we performed formal interaction tests to examine potential effect modifications across subgroups, with the resulting p-values for interaction clearly indicated in the last column of Figure 5 (labeled as "p for trend"). All trend P-values remained > 0.005 after multiple-testing correction. We appreciate your attention to methodological rigor and hope this clarification addresses your concern. Please don't hesitate to let us know if you require any additional information.

*7.Discussion and Interpretation of AUC Values*

*-Temper the language in your conclusions. The ROC AUC for ACAG (0.66–0.67) is modest. Please clarify that ACAG may complement—but not outperform—established tools like SOFA or MELD.*

**The author's response:**

Thank you for your careful and constructive feedback. As suggested, I have revised the discussion section to state: "However, the current evidence suggests that ACAG may serve as a potential supplementary tool rather than outperform well-validated scoring systems such as SOFA or MELD.

***Cleaned*** ***Revised Manuscript, Page 21, lines 324-326:***

In this study, univariate and multivariate analyses were performed on critically ill patients with liver cirrhosis, revealing that the ACAG serves as an independent predictor of both short-term and long-term all-cause mortality in severe liver cirrhosis. Patients were stratified into two groups based on ACAG levels: normal AG and high AG. K-M survival analysis demonstrated significantly worse short-term and long-term prognoses in the high ACAG group. ACAG levels exhibited moderate discriminative ability in predicting mortality at multiple time points in cirrhotic patients. However, the current evidence suggests that ACAG may serve as a potential supplementary tool rather than outperform well-validated scoring systems such as SOFA or MELD. Additionally, RCS analysis identified a linear relationship between ACAG levels and cirrhosis prognosis, indicating that elevated ACAG is associated with poorer outcomes. Subgroup analysis further confirmed the absence of significant interactions between ACAG and other prognostic factors in severe liver cirrhosis, underscoring the reliability of ACAG as a robust prognostic marker.

*8.Revision of Discussion Section*

*-Improve contextualization of your findings with prior literature on cirrhosis-related prognostic tools. Streamline redundant pathophysiology and highlight clinical relevance of ACAG in resource-limited settings.*

**The author's response:**

Thank you for your valuable feedback. I agree that my previous version overemphasized pathophysiological details at the expense of clinical relevance. Below is my revision.

***Cleaned*** ***Revised Manuscript, Page 22-23, lines 347-386:***

The AG is a fundamental parameter for evaluating acid-base disturbances, particularly in diagnosing metabolic acidosis in cirrhosis^[31]^. Progressive hepatic dysfunction in these patients leads to metabolic derangements, including: (1) impaired lactate metabolism—causing systemic accumulation, Type A lactic acidosis (secondary to tissue hypoxia and hemodynamic instability) ^[32, 33]^, and Type B lactic acidosis (due to reduced hepatic perfusion and clearance) ^[34, 35]^, often manifesting as mixed lactic acidosis with elevated AG; (2) impaired ketone regulation, which may precipitate ketoacidosis, especially in diabetics or insulin-deficient individuals^[36, 37]^; and (3) renal insufficiency, a common complication in advanced cirrhosis that exacerbates acidosis^[38]^. These mechanisms underscore the importance of AG monitoring for early detection and intervention^[39]^. Additionally, hypoalbuminemia—a hallmark of cirrhosis—significantly impacts AG as albumin is a major unmeasured anion^[12, 40]^. Conventional AG may underestimate acid-base disturbances in these patients, necessitating ACAG for accurate assessment^[41]^.

The SOFA score, despite its widespread use in intensive care, has limited relevance to cirrhotic patients due to their unique pathophysiology^[42]^. Similarly, the MELD score, though it is the current standard for prognosis, only considers a few biochemical parameters (INR, creatinine, and bilirubin), so it cannot account for critical complications like hepatic encephalopathy, spontaneous bacterial peritonitis, and variceal hemorrhage^[43, 44]^. Recent modifications, such as incorporating serum sodium (MELD-Na) or additional variables like age and albumin (MELD 3.0), have shown improved prognostic performance, underscoring the need for score optimization^[45, 46]^.

The ACAG introduced in this study can mitigate these limitations to some extent. As a robust indicator of acid-base disturbances, ACAG elevation correlates strongly with metabolic acidosis severity, hepatic impairment, and adverse clinical outcomes. By incorporating albumin alongside anion gap measurements, ACAG provides a more comprehensive representation of patient pathophysiology. Importantly, our data show that ACAG enhances mortality risk prediction when combined with either MELD or SOFA scores, offering a valuable advancement in cirrhosis prognostic modeling.

Clinically, ACAG offers several distinct advantages. It enables rapid risk stratification of critically ill cirrhotic patients, facilitating timely clinical decision-making. Elevated values predict adverse outcomes and warrant aggressive intervention, while lower values may support therapeutic de-escalation. This risk stratification promotes resource optimization and personalized treatment. The practical implementation of ACAG is further enhanced by its reliance on routinely available laboratory measurements (albumin and anion gap), eliminating the need for additional testing while maintaining cost - effectiveness and clinical accessibility.

*9.Revision of Conclusions*

*-Please soften your conclusions to reflect limitations inherent in a retrospective single-center database analysis (e.g., causal inference, external generalizability).*

**The author's response:**

Thank you very much for your feedback. Below are my revised conclusions.

***Cleaned*** ***Revised Manuscript, Page 24, lines 400-404:***

This study demonstrates that ACAG levels in cirrhotic patients are significantly associated with both short- and long-term clinical outcomes. Further validation through large-scale, multicenter prospective cohort studies is warranted to confirm these findings and evaluate the broader applicability of ACAG across heterogeneous patient populations.

*10.Validation of X-tile Cutoff*

*-You report an ACAG cutoff of >20 derived via X-tile. Please discuss its clinical applicability and consider a sensitivity analysis (e.g., evaluating model performance across other potential thresholds).*

**The author's response:**

Thank you for your insightful feedback and rigorous critique. The 20 mmol/L cutoff for ACAG was selected based on X-tile analysis and alignment with prior studies. I have already further supplemented the explanation in the original article.

***Cleaned*** ***Revised Manuscript, Page 8, lines 156-159:***

The cutoff value of ACAG was determined to be 20 mmol/L by optimizing 30-day post-admission mortality using X-tile analysis (v3.6.1, Yale University, USA) as well as referring to prior studies^[19-21]^ , with patients being divided into two groups: those with ACAG ≤20 mmol/L and those with ACAG >20 mmol/L. The Cox model was used for univariate and multivariate analyses to identify independent prognostic factors for 30-day mortality in cirrhosis patients. Results were reported as Hazard ratios (HRs) with 95% confidence intervals (CIs). KM curves were plotted, and the log-rank test compared the groups. ROC analysis evaluated the predictive accuracy of ACAG, AG, ALB, SOFA score, and MELD score for mortality.

*-consider a sensitivity analysis(e.g., evaluating model performance across other potential thresholds).*

**The author's response:**

To bolster confidence in our findings, we performed a sensitivity analysis using the optimal cutoff value from the 30-day mortality ROC curve.

***Cleaned*** ***Revised Manuscript, Page 15, lines 227-232:***

To bolster confidence in our findings, we performed a sensitivity analysis using the optimal cutoff value from the 30-day mortality ROC curve. Specifically, we analyzed ACAG at a cutoff value of 18.375. After adjusting for all potential confounding covariates, all P-values across the assessed time points were P < 0.001, underscoring the stability and significance of our results. For further details, see **Supplementary Table 1**.

Supplementary Table1

Association between ACAG and mortality in patients with cirrhosis

| **Outcome** | Unadjusted HR (95%CI) | Model1 HR (95%CI) | Model2 HR (95%CI) |
| --- | --- | --- | --- |
| **30-d** | | | |
| ACAG≤18.375 | 1 | 1 | 1 |
| ACAG＞18.375 | 2.39(2.08, 2.75) | 2.40(2.09, 2.76) | 1.64(1.41, 1.91) |
| P | <0.001 | <0.001 | <0.001 |
| **90-d** | | | |
| ACAG≤18.375 | 1 | 1 | 1 |
| ACAG＞18.375 | 2.26(1.99, 2.57) | 2.27(1.99, 2.58) | 1.51(1.31, 1.75) |
| P | <0.001 | <0.001 | <0.001 |
| **180-d** | | | |
| ACAG≤18.375 | 1 | 1 | 1 |
| ACAG＞18.375 | 2.27(1.99, 2.58) | 2.27(2.00, 2.59) | 1.52(1.32, 1.75) |
| P | <0.001 | <0.001 | <0.001 |
| **365-d** | | | |
| ACAG≤18.375 | 1 | 1 | 1 |
| ACAG＞18.375 | 2.27(2.00, 2.58) | 2.28(2.00, 2.59) | 1.53(1.32, 1.76) |
| P | <0.001 | <0.001 | <0.001 |

Model 1: Adjusted age and Gender.

Model 2: Model 1+ Temperature, Platelet, WBC, Sodium, INR, BUN, Sepsis, Variceal bleeding, SBP, Ascite, HRS, HE, AKI, and CRRT.

### Recommended Revisions (Not Mandatory for Acceptance):

*1.Comparative Prognostic Value*

*-Provide clearer insights into how ACAG might be used alongside MELD and SOFA. A decision curve analysis or net reclassification index could add depth.*

**The author's response:**

We sincerely appreciate the reviewer’s valuable comments. Based on these suggestions, we will revise the manuscript by integrating MELD and SOFA scores with ACAG to better illustrate its clinical utility. We first demonstrated the enhanced predictive ability of our combined scoring approach by adding ROC curves for SOFA+ACAG and MELD+ACAG, which show improved discrimination compared to the individual scores alone. Furthermore, we performed decision curve analysis (DCA) to validate whether the combined model enhances predictive accuracy compared to MELD or SOFA scores individually, and to confirm that the combined models provide greater net clinical benefit than either SOFA or MELD alone. For detailed results, please refer to **Figure 3**, **Supplementary Figure 1**, and **Supplementary Figure 2**.

***Cleaned*** ***Revised Manuscript, Page 15-16, lines 244-262:***

Compared to AG or albumin alone, the ACAG demonstrated better predictive value for both short- and long-term mortality.

For 30-day, 90-day, 180-day, and 365-day mortality predictions, ACAG achieved AUCs of 0.671 (CI: 0.649–0.693), 0.663 (CI: 0.641–0.684), 0.664 (CI: 0.643–0.685), and 0.665 (CI: 0.643–0.686), respectively, consistently outperforming AG (0.651, 0.647, 0.649, 0.649) and albumin (0.541, 0.534, 0.534, 0.534).

The predictive performance was further enhanced when ACAG was combined with SOFA or MELD scores. The SOFA+ACAG model achieved AUCs of 0.704, 0.699, 0.701, and 0.700 for 30-day, 90-day, 180-day, and 365-day mortality, respectively. Similarly, the MELD+ACAG combination demonstrated robust performance, with AUCs of 0.710, 0.709, 0.711, and 0.711, respectively. More details are provided in **Table 3** and **Fig 3**.

The decision curve analysis (DCA) for SOFA versus SOFA+ACAG and MELD versus MELD+ACAG across four follow-up periods (30-day, 90-day, 180-day, and 365-day) is presented in **Supplementary Figures 1** and **2 (SF1** and **SF2).** At all time points, the curves for SOFA+ACAG and MELD+ACAG showed superior net benefit compared to SOFA and MELD alone, respectively. This indicates that incorporating ACAG into the MELD and SOFA scoring systems enhances mortality risk stratification and clinical utility.

*2.Discussion Clarity*

*-The discussion on acid-base physiology, lactic/ketoacidosis, and albumin’s role is scientifically accurate but overly repetitive. Condense and focus on clinical applicability.***The author's response:**

We sincerely appreciate your valuable comments. As suggested, we have condensed the pathophysiological discussion and emphasized the clinical relevance, with detailed changes marked in Revision 8.

***Cleaned*** ***Revised Manuscript, Page 22-23, lines 347-386:***

The AG is a fundamental parameter for evaluating acid-base disturbances, particularly in diagnosing metabolic acidosis in cirrhosis^[31]^. Progressive hepatic dysfunction in these patients leads to metabolic derangements, including: (1) impaired lactate metabolism—causing systemic accumulation, Type A lactic acidosis (secondary to tissue hypoxia and hemodynamic instability) ^[32, 33]^, and Type B lactic acidosis (due to reduced hepatic perfusion and clearance) ^[34, 35]^, often manifesting as mixed lactic acidosis with elevated AG; (2) impaired ketone regulation, which may precipitate ketoacidosis, especially in diabetics or insulin-deficient individuals^[36, 37]^; and (3) renal insufficiency, a common complication in advanced cirrhosis that exacerbates acidosis^[38]^. These mechanisms underscore the importance of AG monitoring for early detection and intervention^[39]^. Additionally, hypoalbuminemia—a hallmark of cirrhosis—significantly impacts AG as albumin is a major unmeasured anion^[12, 40]^. Conventional AG may underestimate acid-base disturbances in these patients, necessitating ACAG for accurate assessment^[41]^.

The SOFA score, despite its widespread use in intensive care, has limited relevance to cirrhotic patients due to their unique pathophysiology^[42]^. Similarly, the MELD score, though it is the current standard for prognosis, only considers a few biochemical parameters (INR, creatinine, and bilirubin), so it cannot account for critical complications like hepatic encephalopathy, spontaneous bacterial peritonitis, and variceal hemorrhage^[43, 44]^. Recent modifications, such as incorporating serum sodium (MELD-Na) or additional variables like age and albumin (MELD 3.0), have shown improved prognostic performance, underscoring the need for score optimization^[45, 46]^.

The ACAG introduced in this study can mitigate these limitations to some extent. As a robust indicator of acid-base disturbances, ACAG elevation correlates strongly with metabolic acidosis severity, hepatic impairment, and adverse clinical outcomes. By incorporating albumin alongside anion gap measurements, ACAG provides a more comprehensive representation of patient pathophysiology. Importantly, our data show that ACAG enhances mortality risk prediction when combined with either MELD or SOFA scores, offering a valuable advancement in cirrhosis prognostic modeling.

Clinically, ACAG offers several distinct advantages. It enables rapid risk stratification of critically ill cirrhotic patients, facilitating timely clinical decision-making. Elevated values predict adverse outcomes and warrant aggressive intervention, while lower values may support therapeutic de-escalation. This risk stratification promotes resource optimization and personalized treatment. The practical implementation of ACAG is further enhanced by its reliance on routinely available laboratory measurements (albumin and anion gap), eliminating the need for additional testing while maintaining cost - effectiveness and clinical accessibility.

3. *Minor Language Editing*

*-The manuscript would benefit from a thorough English language edit to address minor grammar, punctuation, and typographical issues.*

**The author's response:**

Thank you for your valuable suggestion. We appreciate your attention to the clarity of our manuscript. In response, we have carefully revised the entire manuscript to improve its grammar, punctuation, and overall language quality. We also sought assistance from a native English speaker to ensure that the text meets academic standards. We hope the revised version is now clearer and more polished.
